# A Recent Systematic Increase in Vapor Pressure Deficit over Tropical South America

**DOI:** 10.1038/s41598-019-51857-8

**Published:** 2019-10-25

**Authors:** Armineh Barkhordarian, Sassan S. Saatchi, Ali Behrangi, Paul C. Loikith, Carlos R. Mechoso

**Affiliations:** 1 Department of Atmospheric and Oceanic Sciences, University of California, Los Angeles, USA; 2 Jet Propulsion Laboratory, California Institute of Technology, Pasadena, USA; 3 Institute of Environment and Sustainability, University of California, Los Angeles, USA; 4 University of Arizona, Department of hydrology and atmospheric sciences, Tucson, USA; 5 Portland State University, Department of Geography, Portland Oregon, USA

**Keywords:** Climate sciences, Hydrology

## Abstract

We show a recent increasing trend in Vapor Pressure Deficit (VPD) over tropical South America in dry months with values well beyond the range of trends due to natural variability of the climate system defined in both the undisturbed Preindustrial climate and the climate over 850–1850 perturbed with natural external forcing. This trend is systematic in the southeast Amazon but driven by episodic droughts (2005, 2010, 2015) in the northwest, with the highest recoded VPD since 1979 for the 2015 drought. The univariant detection analysis shows that the observed increase in VPD cannot be explained by greenhouse-gas-induced (GHG) radiative warming alone. The bivariate attribution analysis demonstrates that forcing by elevated GHG levels and biomass burning aerosols are attributed as key causes for the observed VPD increase. We further show that There is a negative trend in evaporative fraction in the southeast Amazon, where lack of atmospheric moisture, reduced precipitation together with higher incoming solar radiation (~7% decade^−1^ cloud-cover reduction) influences the partitioning of surface energy fluxes towards less evapotranspiration. The VPD increase combined with the decrease in evaporative fraction are the first indications of positive climate feedback mechanisms, which we show that will continue and intensify in the course of unfolding anthropogenic climate change.

## Introduction

Earth’s climate system changes over time due to the influence of two fundamentally different mechanisms: (1) chaotic/stochastic interactions within climate system components (e.g., atmosphere/ocean natural modes of variability, such as the El Nino-Southern Oscillation (ENSO) or Atlantic Multidecadal oscillation (AMO)), and (2) changes in the planet’s energy budget due to factors external to the system. The externally forced component can be subdivided into anthropogenic forcing (e.g., greenhouse gases, anthropogenic aerosols) and natural external forcing (e.g., solar forcing and stratospheric aerosols due to large volcanic eruptions).

Tropical South America (TSA), with a large intact rainforest region is an excellent laboratory to examine the changes of the climate system due to its significant role in both global carbon and water cycles, and strong feedback mechanisms that can potentially exacerbate changes in climate^1,2^. In future climate change scenarios TSA is a drought hotspot due to its high sensitivity to warming and drying signals^3^. The region has already experienced large scale and frequent episodic droughts in association with ENSO events and other severe climate anomalies^3–8^. The 2005 and 2010 extreme drought events over Amazonia led to the most negative annual carbon balance ever recorded in the region^9,10^. After the 2005 mega-drought, a significant loss of carbon over the entire Amazon basin has been detected and continued persistently until 2008^11,12^. The recurrent droughts over Amazonia during the 2005 to 2016 period have been slowing down recovery of the region’s hydrological system and enhancing wildfire risk and tree mortality^13,14^. The recent El Nino-associated 2015–2016 drought cannot be explained by internal ocean-atmosphere climate variability alone, and external drivers have likely provided a strong contribution^15^.

In addition to the global manifestation of greenhouse gas (GHG) forcing, the most important external drivers of climate change over TSA are the effects of black carbon aerosols released from biomass burning in the Amazon and Savanah regions^16^ and the changes in land-use/land-cover due to expanding agriculture activities^16,17^. The external forcing of climate change over TSA is therefore, influenced by changes in greenhouse gases (GHGs), anthropogenic aerosols (AA) and land-use/land-cover change (LU). The aim of this study is to distinguish the internally generated changes in SA climate from those that are externally and systematically forced. We use the output of multi-model ensemble runs from Phase 5 of the Coupled Model Inter-comparison Project^18^ (CMIP5) to assess the response of TSA climate to each of these drivers in isolation and quantify their contribution to the observed trends over the region. We note that changes in the physiological effects of CO_2_ may also be an important external driver of climate change in SA, but we will not consider them in isolation from the radiative effect of CO_2_ in the present study.

Any changes in the physiological function of the forest depend strongly on the atmospheric demand for water, which is driven by two factors: Vapor Pressure Deficit (VPD; saturation minus actual water vapor pressure) and net radiation. The predicted increase of atmospheric demand for water due to global warming^19^ will cause droughts to become more widespread and severe^20^. A recent study based on statistical analysis demonstrated that the dry seasons (June-October) over tropical SA are getting warmer under the influence of anthropogenic climate change^21^. This is occurring in conjunction with a positive trend in incoming solar radiation in late dry season, which can partly be attributed to cloud reduction due to radiatively absorbing aerosols such as black carbon^21^. The enhanced incoming solar radiation, together with the global manifestation of increased concentrations of GHG results in regional amplification of daily maximum temperature over tropical SA^21^. Thus, as the climate continues to warm over the region, further drying over land is expected^22^. This results in greater potential evapotranspiration over land compared to the ocean^19^, and in land-atmosphere feedbacks that amplify the increase of aridity over land^23^. On the supply side of the water balance, the reduction of dry season rainfall over the south and southeastern Amazon in the recent decades has been determined to be well beyond the ranges expected from natural internal variability of the climate system^24^. The detected “drier dry season” was attributed to simultaneous effects of increasing GHG concentrations and changes in LU over 1983–2012 time period^24^.

In the present paper we focus on VPD, which is an accurate measure for predicting plant transpiration and water loss^25^. By combining relative humidity and temperature into a single quantity, VPD is a key factor controlling evaporative demand [Penman’s equation^26^] and carries several features in the context of fire and drought analysis^25,27^. Therefore, it is important to assess changes in VPD over SA and relate the detected changes to external drivers of climate change, an effort that is referred to as “attribution analysis”^28,29^. The principle behind the present attribution analysis is to assess the amplitude of the response of VPD to each external forcing (GHG, AA, LU) from the observations via estimation of scaling factors.

## Results

### Long-term trends in observed VPD data

**Figure 1:**
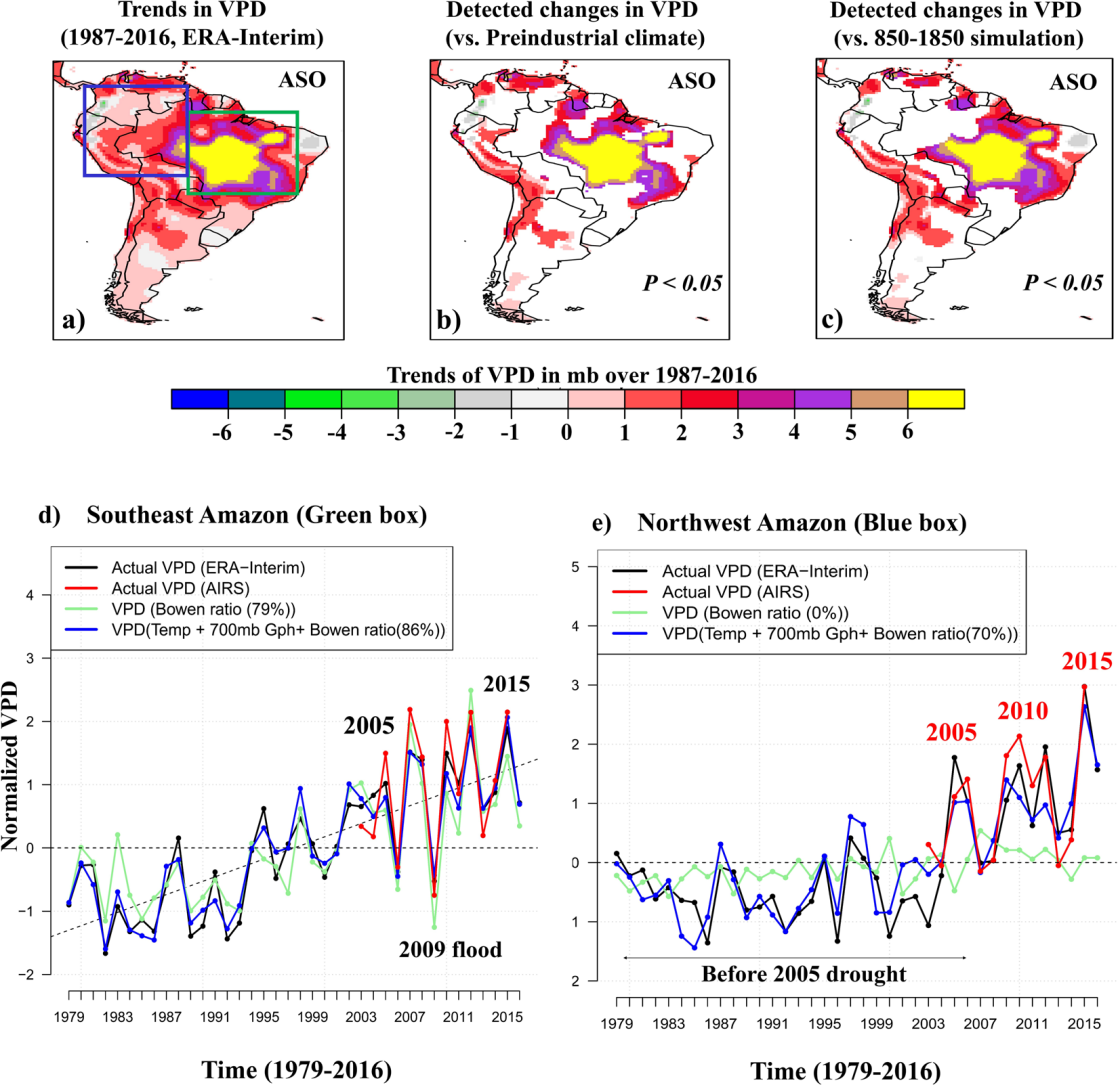
*Top:* Detection of externally forced changes in VPD trends. *Bottom:* The effects of land surface and atmospheric conditions on VPD. (**a**) Trends in VPD derived from ERA-I during 1987–2016 in dry (ASO) months. (**b**) Regions where externally forced changes of VPD are detectable (in compare with 400 pseudo-realizations of unforced trends derived from 12,000-year Pre-industrial simulations). (**c**) Regions where anthropogenically forced changes of VPD are detectable (P < 0.05) in ASO (in comparison with 30 pseudo-realizations of naturally forced trends derived from 850–1850 simulations). *Bottom:* The time series of actual normalized VPD (i.e., minus mean and divided by the standard deviation) in ASO based on ERA-I reanalysis data (1979–2016, black), AIRS satellite data (2003–2016, red), and reconstructed VPD via linear regression model (**d**) over the southeast Amazon, (**e**) the northwest Amazon. The linear regression model is based on the Bowen ratio (proxy for energy partitioning), temperature (proxy for warming) and 700-mb geopotential height (proxy for large scale moisture transport). The percent variance explained by the regression model is noted.

We calculate VPD by using monthly near surface air temperature and dew point from the ERA-Interim (ERA-I) reanalysis dataset for the 30-year period 1987–2016, and from the Atmospheric Infrared Sounder (AIRS) for the period 2003–2016 (*see Methods*). Figure 1a displays the spatial variations of the linear trend in VPD from ERA-I corresponding to the peak of dry months (August-October, ASO, Fig. S6). During ASO, VPD shows an increasing trend over the southeastern Amazon with values of ~6 ± 2 mb over the period 1987–2016, which results from ~+2 °C warming (saturation vapor pressure, *e*_*s*_) and ~−2.5 °C decrease in dew point (actual vapor pressure, *e*_*a*_). Additional estimation using AIRS satellite data also captures an increasing trend of VPD (decreasing trend in relative humidity) over the period of observation (2003–2016), which is most pronounced over the eastern TSA and larger during the daytime than nighttime (Supp. Figs S1 and S2). The fifth generation of ECMWF atmospheric reanalysis data (ERA5)^30^, further confirms the observed increasing trend of VPD over the region (Supp. Fig. S3).

The time series of the normalized VPD (i.e., minus mean and divided by the standard deviation) based on the ERA-I and AIRS satellite show gradually increasing values over the southeast Amazon, with a detectable (P < 0.05) linear trend (Fig. 1d). Over the northwest Amazon, however, the increase is not linear but punctuated by episodic events representing major droughts (2005, 2010, 2015) (Fig. 1e). The 2005 mega drought^7^ brought the atmospheric demand for water (positive VPD anomalies) to a higher level. Particularly, the anomalously high VPD during the 2015 drought is the highest in the record since 1979 (Fig. 1e), which points to an intensification of atmospheric drying in the region.

### Detection of systematically forced changes in VPD

To assess whether the observed increasing trends in VPD, over the last three decades are externally and systematically forced, we compare them with estimates corresponding to the natural variability of the climate system. To obtain these estimates we use long pre-industrial control simulations from global climate models (GCMs) participating in the CMIP5 project. The simulations we use are performed under control conditions (i.e., with constant atmospheric composition, no episodic volcanic influences, and no variation in solar output). Since climate models may underestimate variability, we double the simulated variance prior to the attribution analysis^31^. The 12,000-year pre-industrial control (PIC) runs, which are the concatenated PIC runs of 19 models, provide up to 400 pseudo-realizations of how the climate might have changed in the absence of external influences. In addition, we use the Paleo simulations over the 0850–1850 millennium derived from CCSM4 model to obtain an estimate of natural (internal + external) variability of VPD. These millennium simulations provide 30 pseudo-realizations of how VPD might have changed in the absence of anthropogenic influences. In this manner, we can test the null-hypothesis that the observed trend in VPD is within the 5–95^th^ percentile distribution of unforced trends (as derived from the pre-industrial control simulations) or naturally forced trends (as derived from the 850–1850 millennium simulation).

The results displayed in Fig. 1b indicate that systematically forced changes are detectable (<5% risk of error) in observed increasing trends of VPD in ASO over the southeast Amazon. This result remains robust after comparison with the trends obtained with naturally forced changes derived from the 0850–1850 simulation (Fig. 1c). In the wet months (March-May), however, a substantial portion of VPD variability can be explained by the natural variability of the climate system (Supp. Fig. S4). We note here the adoption of a risk of false rejection (<5%) of the null hypothesis of “no external forcing”. Since when the regional null hypothesis is valid, on an average *n* = *0*.*05* *m* local alternatives will falsely be rejected (m number of grid points)^32^.

### Response of VPD to external climate drivers

The climate over TSA is potentially influenced by three external forcings: greenhouse gases (GHGs), anthropogenic aerosols (AA) and land-use/land-cover change (LU). In this section we assess the response of VPD to these forcings in isolation by using multi-model ensemble mean single forcing experiments from the CMIP5 archive, such as AA-forcing only, GHG-forcing only and LU-forcing only. We further subdivided the AA simulations into two groups to separately investigate the impact of aerosol concentration on the cloud albedo^33^ (“first indirect effect”), and the cloud lifetime^33^ (“second indirect effect”). Hence, AA1 simulations include both the first and second indirect effect of areoles on clouds, while AA2 simulations include the first indirect effect only.

**Figure 2:**
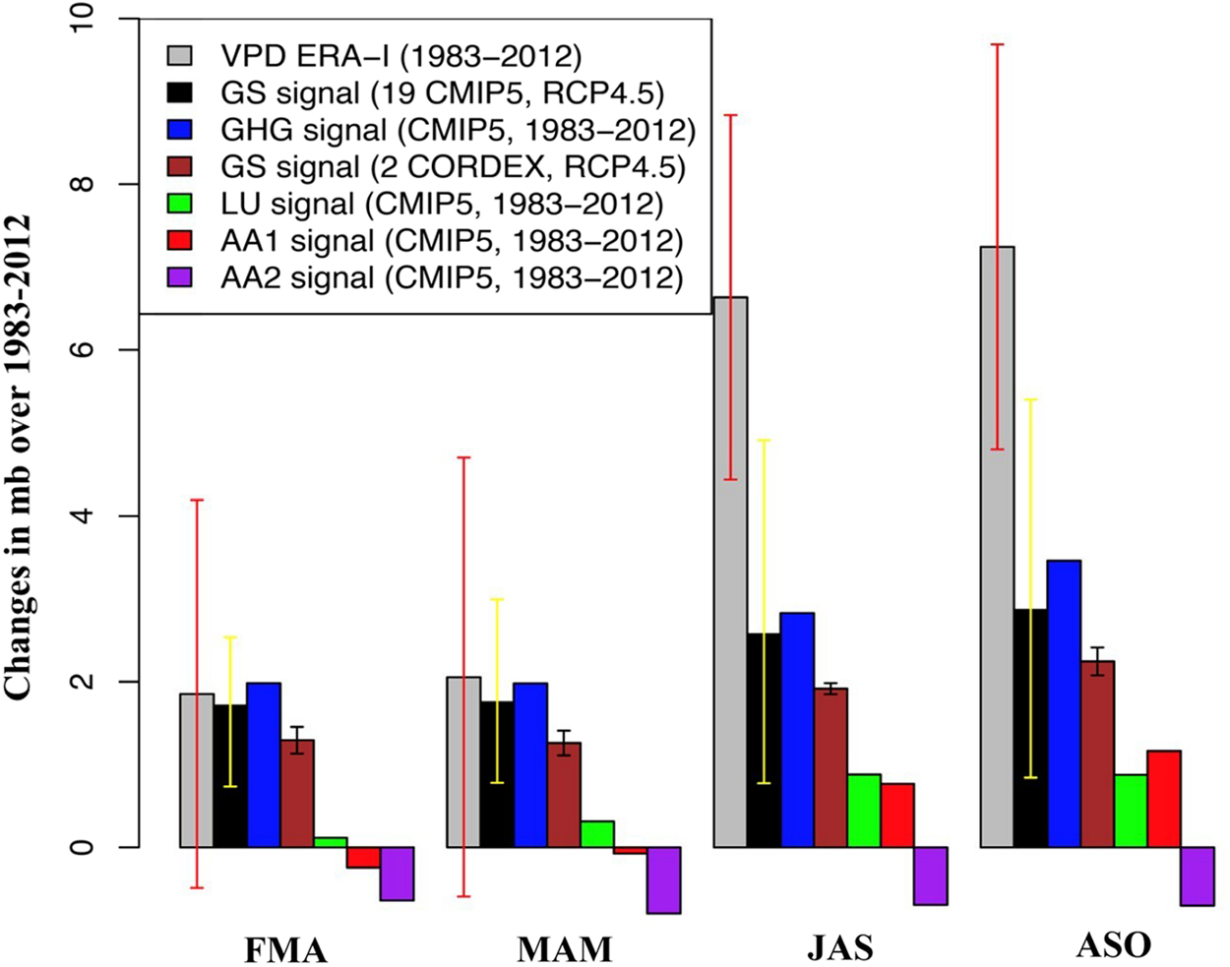
Area mean change of VPD over southeast Amazon (green box in Fig. 1a) in comparison with the response of VPD to external climate drivers. Observed 30-year trends in VPD over southeast Amazon from 1983 to 2012 (mb over 30-years) for sliding 3-month windows (grey bars) in comparison with GS signal (RCP4.5 scenario) derived from 19 GCMs of CMIP5 (black bars), GS signal (RCP4.5 scenario) derived from 2 RCMs of CORDEX (brown bars), historical greenhouse-gas signal derived from multi-model ensemble mean (GHG, blue bars), Land-use change signal (LU, green bars), aerosols signal with (AA1, red bars), and without (AA2, purple bars) the “cloud lifetime effect” of aerosols. The red whiskers indicate the 95%-tile uncertainly range, derived from model-based estimate of natural (internal) variability (400 pseudo-realizations of unforced trends derived from 12,000-year Pre-industrial simulations). The whiskers on the black and brown bars show spread of trends of 19 GCMs and 2 RCMs, respectively. The blue, green, red and purple bars are derived from multi-model ensemble mean single forcing experiments. Externally forced changes are detectable in observed VPD trend (grey bars) where the red whiskers exclude zero.

Figure 2 shows the area average of 30-year trends in VPD over southeastern Amazon for wet (FMA, MAM) and dry (JAS, ASO) months. We considered the 95%-tile internal variability-generated uncertainly range (red whiskers) derived from model-based estimate of natural (internal) variability (*See Method*). The linear trend of VPD over southeastern Amazon has a notable seasonal cycle with maximum increases (minimum variances) in dry months and minimum increases (maximum variances) in wet months.

The VPD has an increasing trend in climate simulations in response to historical well-mixed GHG forcing, Greenhouse gas and Sulfate aerosols (GS) forcing based on RCP4.5 scenario derived from 19 global climate models (GCMs) and 2 regional climate models (RCMs) (blue, black and brown bars in Fig. 2, respectively). The LU-forcing only simulations show an increase in VPD. As shown in Fig. 2, VPD tends to increase during the dry months in response to LU forcing, while in wet months the response is almost negligible (green bars in Fig. 2). In the tropics, the net impact of LU is typically a non-radiative warming (biophysical effect) due primarily to decreasing evapotranspiration (ET) and surface roughness^34,35^.

The negative trend in VPD from aerosol-only AA2 simulations without the “cloud lifetime effect” of aerosols (purple bars in Fig. 2) contradicts the small positive trends produced in the AA1 simulations that consider the “cloud lifetime effect” of aerosols (red bars in Fig. 2). This contradiction indicates that the direct aerosol–radiation interactions results in a net cooling^36^. However, radiatively absorbing aerosols such as black carbon can warm the atmosphere and surface through re-emitted longwave radiation^37^ and in turn decrease the cloud cover at and above the altitude where it is present^38^, producing a warming that counteracts the direct aerosol cooling^36^. The results in Fig. 2 are reasonable since the main source of anthropogenic aerosols over tropical SA in dry months is black carbon aerosol (BC) due to biomass burning^39^ (agricultural waste burning and forest fires).

It is notable that in the wet months the GHG-induced radiative warming can exclusively explain the observed VPD increase (Fig. 2). However, in dry months the simulated (projected) trends in VPD as response to GHG (GS) are much smaller than those observed. Thus, the GHG-induced warming alone cannot explain the observed atmospheric drying in dry months and other local forcing, such as AA1-induced and LU-induced local warming and drying (because in dry conditions over land much of that heat goes into drying^22^) are also at work. It is interesting to note that the strongest response of VPD to AA and LU is shown in late dry months (ASO).

### Univariant signal attribution

**Figure 3:**
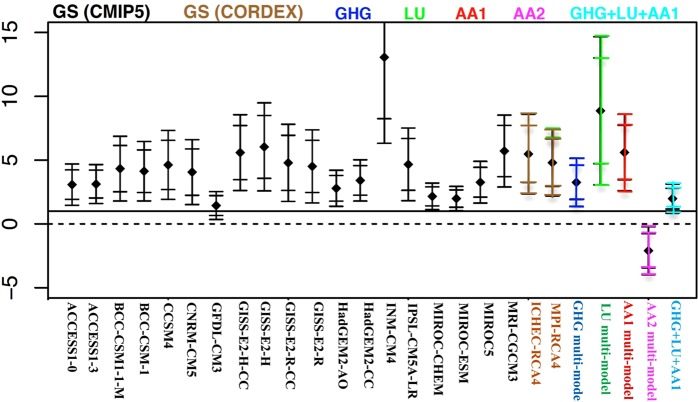
One-dimensional (univariant) attribution over southeast Amazon. Scaling factors of observed VPD changes in ASO against 19 global GS signals (CMIP5) based on the RCP4.5 scenario (black bars), the regional GS signal derived from CORDEX (brown bars), the historical greenhouse-gas signal (GHG, blue bars), the land-use change signal (LU, green bar), the aerosols signal with (AA1, red bar) and without (AA2, purple bar) the “cloud-lifetime effect”, the combined signal from GHG, AA and LU (light blue bar). The whiskers show the 95^th^ %-tile range of internal variability-generated uncertainties associated with scaling factors for the raw and double the model variance, derived from 12,000-year control simulations. Attribution is claimed in cases where the scaling factors are inconsistent with zero but consistent with one.

In this section, we attribute the detected changes of VPD over 1983–2012 (CMIP5 simulations are till 2012) in ASO to external drivers of climate change, such as GHG, AA1, AA2 and LU forcings. Since in wet months (MAM) a substantial portion of VPD variability can be explained by the natural variability of the climate system (Fig. S4), the following attribution analysis is focused on dry ASO months where the observed trends in VPD are found to be larger than natural variability-generated trends and need to be explained by external drivers. Our attribution analysis is based on assessing the amplitude of the response of VPD to each external forcing from the observations via the estimation of scaling factors^21,24^ (*See Methods*). The null hypothesis is that the observed change in VPD is drawn from a hypothetical population of a climate disturbed by a specific external influence. Figure 3 displays the scaling factors that result from the one-dimensional attribution analysis in which observed VPD changes over the southeastern Amazon are projected onto the response patterns to GS derived from both GCMs and RCMs, along with historical well-mixed GHG, AA1, AA2 and LU forcing. The whiskers show the 95^th^ %-tile range of internal variability-generated uncertainties associated with scaling factors in an undisturbed climate based on 400 control segments, for the raw and double the model variance. Attribution is claimed when the scaling factors are inconsistent with zero but consistent with one (i.e., they don’t cross the zero line but do cross the one line).

According to Fig. 3, the range of the internal variability-generated uncertainty of scaling factors does not include the zero line for all 19 global GS signals (black bars), the 2 regional GS signals (brown bars) and the historical well-mixed GHG signal (blue bar). This indicates that irrespective of the models used (with different sensitivities to the forcing) the elevated GHG concentration has a robust detectable influence on the observed VPD increase (<5% risk of error). Neither the scaling factors of the AA1 signal (red bar) nor the LU signal (green bar) include the zero line, suggesting that the local warming/drying induced by AA1 and LU forcings also contribute significantly to the observed increase in atmospheric demand for water over the region.

Aerosols-only forcing simulations without the “cloud lifetime effect” (AA2) display negative regression indices (purple bar in Fig. 3), indicating that including the effect of aerosols on cloud lifetime yields results that are more compatible with the observed record.

Notably, the range of internal variability-generated uncertainty derived from the LU simulations is considerably larger than the range estimated for GHG forcing. This could imply that the signal of VPD increase is dominated by the GHG radiative response, which makes it difficult to separate the smaller and non-radiative LU contribution from the internal climate variability. Results are robust against doubling the internal variability range. In the next step, in order to separate the driver’s contributions to the response a combined influence of GHG, AA1 and LU should be considered. In this case a bivariant attribution analysis is required, where the observed trend is projected onto two hypothetical signals simultaneously.

### Bivariate signal attribution

**Figure 4:**
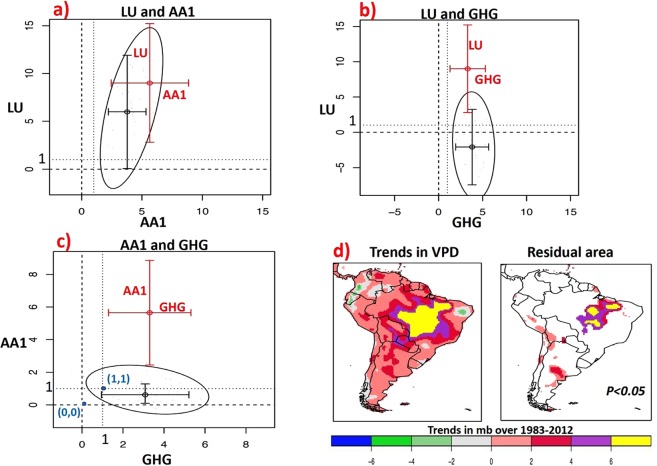
Two-dimensional (bivariant) attribution over the southeast Amazon and Residual area (incomplete attribution). The ellipses display the 90% of the estimated joint distribution of scaling factors for the (**a**) AA1&LU, (**b**) GHG&LU, (**c**) GHG&AA1 signals when observed data are regressed onto two signals simultaneously during 1983–2012. The black and red whiskers indicate the bivariate and univariate 1-dimensional 95^th^ %-tile intervals of the internal variability-generated uncertainty for the two signals, respectively. Bivariant attribution is claimed in cases where the ellipse excludes the origin (0, 0) but the point (1, 1) lies inside the ellipse. (**d**) Residual area (incomplete attribution): Regions in Brazil’s “arc of deforestation” where externally forced changes are still detectable after removing the effect of GHG, LU and AA1 forcing (at 5% level).

To separate the contribution of each driver to the VPD response, we perform a bivariate (two dimensional) attribution analysis. Accordingly, the observed data are projected onto the response patterns of VPD to AA1&LU (Fig. 4a), GHG&LU (Fig. 4b) and GHG&AA1 (Fig. 4c) forcings simultaneously. The black and red whiskers in Fig. 4 indicate the bivariate and univariate one-dimensional 95^th^ %-tile intervals of the internal variability-generated uncertainties for the two hypothetical signals, respectively. The bivariate two-dimensional uncertainty contour of scaling factors for the two signals are shown with an ellipse. Two-dimensional attribution is claimed in cases where the ellipse excludes the origin (0, 0) but the point (1, 1) lies inside the ellipse.

In Fig. 4a, the ellipse containing 90% of the estimated joint distribution of scaling factors for AA1&LU signals excludes the origin (0, 0), indicating that the effect of AA1 and LU signals are detectable simultaneously. However, the uncertainty range derived from the LU simulations is considerably larger than the range estimated for AA1, indicating that the LU contribution is smaller than the AA1. In the case of the bivariate attribution with AA1&GHG signals (Fig. 4c) the ellipse excludes the origin (0, 0) and the scaling factors are consistent with unit amplitude since the point (1, 1) lies inside the ellipse. Therefore, we conclude that the GHG-induced warming effect and the AA1-induced local warming/drying effect are the dominant external drivers of the observed VPD increase during 1983–2012 over southeastern Amazon, followed by a smaller LU contribution. This result is corroborated by recent observations of large reduction of precipitation in the eastern Amazon attributed to the presence of Black Carbon during the dry season, and the physiological effects of the increasing CO_2_ in the atmosphere causing the reduction of stomatal conductance and the evapotranspiration^40^.

### Residual area (Incomplete attribution)

To assess the completeness of our attribution study, we derive the unexplained components at the local scale. For this, we subtract the attributed changes in VPD from the observed changes and examine the existence of a remaining external forcing. This is done univariately, i.e., for all grid points separately. We examine if the remaining trends are larger than could be expected from natural variations (as provided by the control simulations). Figure 4d shows region were systematically forced changes are still detectable in the remaining trend in VPD after removing the effect of GHG, AA1 and LU. This region aligns with the Amazonian “arc of deforestation”^41,42^, extending from the states of Para and Maranhão in the east to Mato Grosso in the south of the Amazon, where the majority of land use changes have occurred in recent decades. The result may also imply that there is a nonlinear interaction among the impacts of the three drivers across the “arc of deforestation” that leads to an intensification of atmospheric drying.

### Relation of VPD to surface fluxes

As an indicator of land-atmospheric interaction, we inspect the changes of the Evaporative Fraction (EF), which is the ratio of Evapotranspiration (ET) to surface available energy. EF measures the surface energy partitioning towards ET and reflects ecosystem stress, and the physiological effect of CO_2_ on plants^43,44^.

**Figure 5:**
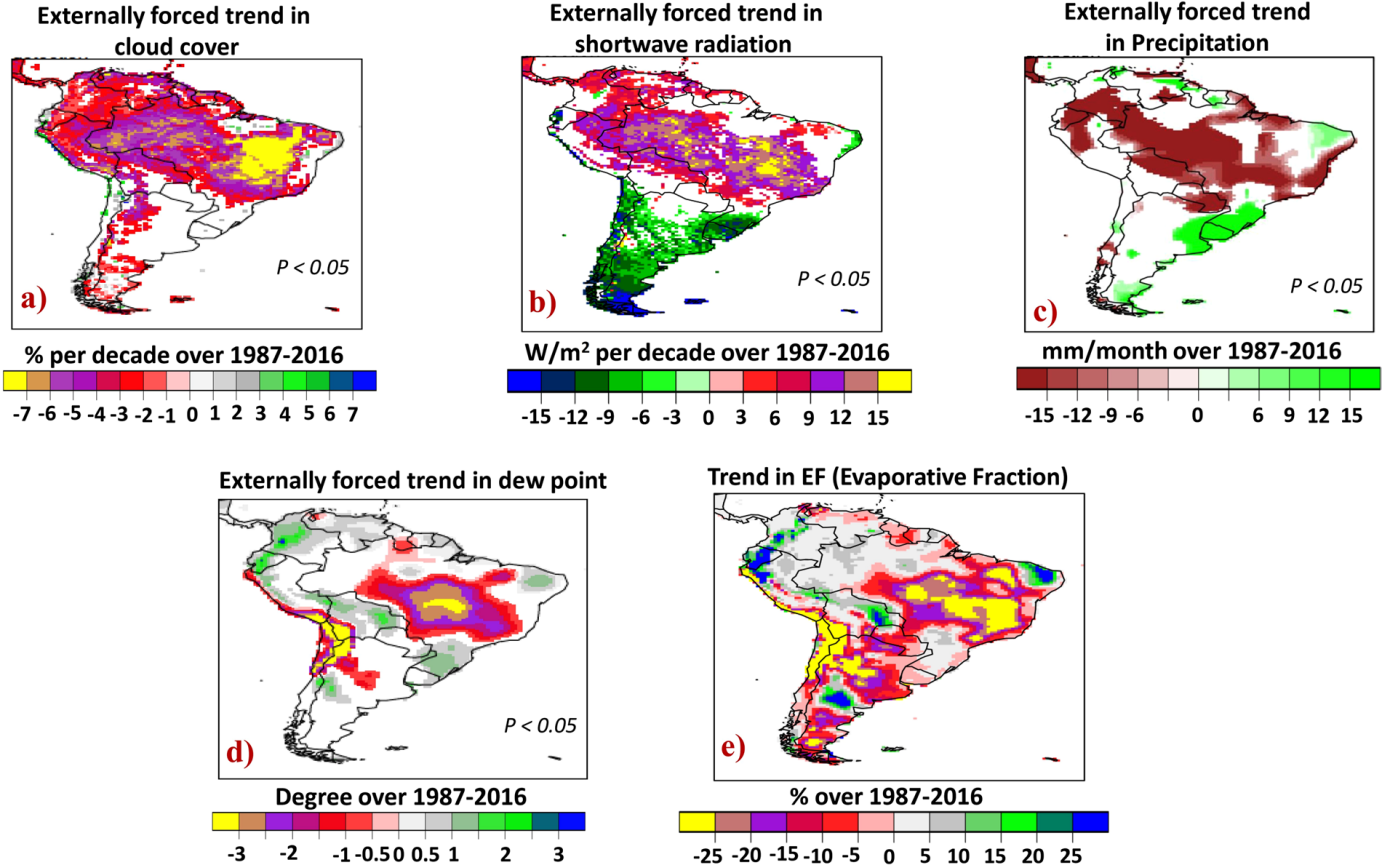
Long-term trend in surface fluxes and parameters over 1987–2016. (**a**) Externally forced trend in total could cover in comparison with 400 pseudo-realizations of unforced trends derived from 12,000-year Pre-industrial simulations, (**b**) in downwelling shortwave radiation, (**c**) in precipitation and (**d**) in dew point. The trend detection is with less than 5% risk of error. (**e**) Trends in Evaporative Fraction (EF) over 1987–2016.

The EUMETSAT satellite data shows a ~7% per decade decreasing trend in cloud cover during ASO over 1987–2016 (Fig. 5a). High-cloud-cover reduction is the major contributor to the observed decline in total cloud fraction (Sup Fig. S9). The decrease in cloudiness goes together with a ~15 W/m^2^/decade increase in downwelling solar radiation based on the EUMETSAT satellite data (Fig. 5b). These negative and positive trends in cloud cover and downwelling shortwave radiation, respectively, are found to be externally and systematically forced (with less than 5% risk of error) because they are beyond the range of trends due to natural variability of the climate system (as obtained in 400 pseudo-realizations of unforced trends derived from 12,000-year Pre-industrial simulations). The GPCP merged station and satellite data also shows an externally forced (P < 0.05) decreasing trend in precipitation during ASO, more than ~15 mm/month over the 1987–2016 time period throughout tropical SA (Fig. 5c). These results are in line with the recently detected externally forced “drier dry season” feature over the region^24^.

The strongest negative trends in EF (Fig. 5e) are found over southeast Amazon (~20% decrease during 1987–2016), where lack of atmospheric moisture (~−2.5 °C decrease in dew point (Fig. 5d)), reduced precipitation (~15 mm/month decrease) together with more incoming solar radiation (~+15 W/m^2^/decade increase) decreases EF and causes more warming as the sensible heat rises (reduction in evaporative cooling) to balance the enhanced incoming solar radiation. The small positive trend observed in EF over areas of northwest Amazon during ASO months may imply the ability of the tropical dense forests with frequent precipitation to maintain high ET under increasing solar radiation^45^.

### Relation of VPD to atmospheric conditions

In order to assess the effects of land surface and atmospheric conditions on VPD, we conducted a multi-linear regression of VPD, temperature, Bowen ratio (sensible heat flux divided by latent heat flux) and 700 mb geopotential height (*See Methods*).

To represent the land-atmosphere interactions we take the Bowen ratio as a measure for energy partitioning, and for the atmospheric circulation we choose the geopotential height at 700 mb where moisture transport is maximized. Figure 1d,e display the actual and reconstructed VPD timeseries based on ERA-I and AIRS satellite data, over southeast and northwest Amazon, respectively. Results suggest that the timeseries of area averaged VPD over southeastern Amazon reconstructed based on the Bowen ratio alone is reasonably accurate. In ASO, about 79% of the VPD variability (R^2^ = 0.88) can be explained by lack of surface moisture (energy partitioning towards less ET). In contrast, over the northwest Amazon rainforest, land surface fluxes play a negligible role and about 70% of the VPD variance can be explained by the GHG-induced warming effect and lack of moisture transport by the large-scale atmospheric circulations. This suggests that increasing VPD in moist tropical forests due to warming can cause drought conditions even if soil moisture does not decrease^46^.

## Discussions

We provided evidence that the magnitude of the positive trends in VPD over the southeastern Amazon during the dry months exceeds the estimated range of trends due to natural variability of the climate system defined in both the undisturbed Preindustrial climate and the 850–1850 millennium. While over the southeast Amazon the enhanced atmospheric drying is systematically forced (p < 0.05), over the northwest the trend in VPD is more of a drought driven episodic increase. The 2015 mega drought brought the atmospheric demand for water (positive VPD anomalies) to its highest level on record since 1979. Our results suggest that the entire TSA region is experiencing a drying of the atmosphere with different regional patterns increasing the demand on tropical forests for water exchange particularly during the dry season.

Reanalysis data already show a trend towards less EF, which is the ratio of ET to surface available energy, over the southeast Amazon. A linear regression analysis show that about 79% of the VPD variability can be explained by lack of surface moisture (energy partitioning towards less ET). These surface flux changes towards less latent heat flux (ET), can significantly delay the wet season onset over the region under an ET-initiated onset mechanism^47,48^. It is to be noted, however, that the supply of ET depends on factors other than just soil moisture such as biomass (leaf area), plant transpiration, and the opening of stomata^49^. Thus, the observed decrease in EF over the southeast Amazon could also be due, at least partially, to the physiological effect of CO_2_^50,51^, as plants are capable of reducing ET in response to increased VPD (high atmospheric demand for water) by closing their stomata, in an effort to conserve water^52^.

A bivariant signal attribution analysis demonstrates that GHG-induced radiative warming, and AA1-induced local warming/drying (due to at least in part to radiatively absorbing BC aerosols that leads to a general decrease of high cloud cover through reductions in dew point), are the dominant external drivers of the VPD increase, followed by a smaller contribution of LU change-induced non-radiative warming (biophysical effect) (at 5% significant level). Results are robust against doubling the internal variability range. It is important to note that the biomass burning is part of land-use change (deforestation) activities. Black Carbon has also been found to drive reduced precipitation over the Amazon is dry season, when higher levels of biomass burning occur, due to temperature-driven circulation change^40^.

The southeast Amazon where continental evapotranspiration (land surface latent heat flux) provides an important moisture source for wet season onset is vulnerable to “drier^24^ and longer^48^ dry seasons” and this vulnerability is exacerbated by land-use change activities (biomass burning and deforestation) in recent decades. This effect is further exacerbated through increasing physiological effects of CO_2_, causing the closure of stomata, reduction of evapotranspiration, and consequently less moisture to fuel the rainfall^40^. Recent mega droughts in the Amazon (2005, 2010, and 2015) have intensified the forests atmospheric aridity and the slow recovery of the region’s hydrological system^11,12^.

Increasing VPD and radiation (~15 W/m^2^ decade^−1^ increase) will naturally increase the demand for photosynthesis and transpiration. However, with water deficit in the soil from reduced precipitation (~−15 mm/month decrease) during the dry season, the probability of plants frequently experience stress that lead to mortality from physiological and hydraulic failure, will increase^53^. In addition, increasing VPD could reduce forest CO_2_ uptake^54^. This may be a strong indication of changes of forest function due to drier atmosphere and seasonal moisture availability.

What is the implication of these results for future changes in VPD? We have shown that the effect of GS signal based on RCP4.5 scenario derived from 19 GCMs and 2 RCMs already has a detectable influence (at 5% significant level) in the recently observed VPD increase. Thus, the currently observed enhanced evaporative demand over the Amazon basin can be used as an illustration of plausible future expected change in the region, which is critical for any mitigation plans and adaptation strategies.

## Methods

### Observation data

We used temperature and relative humidity observations from the Atmospheric Infrared Sounder (AIRS^55^; AMSU), available over the 2003–2015 time period. Since long-term observational products of humidity were not available over northern SA, we used the ERA-Interim reanalysis^56^ data set. This includes adaptive and fully automated bias corrections of satellite observations over the 1979–2017 time period that performed better than other reanalysis data sets over our region of interest^57^. Surface latent and sensible heat fluxes needed to calculate EF (evaporative fraction, the ratio of ET to surface available energy) and the Bowen ratio (the ratio of sensible heat to latent heat fluxes) were obtained from the ERA-Interim dataset. Observational record for precipitation is from the monthly satellite-based Global Precipitation Climatology Project (GPCP) gridded data set [version 2.3]^58^ for the years 1979–2017. To estimate atmospheric circulation variability, we used geopotential height from NCEP-NCAR reanalysis^59^. The cloud cover data and surface incoming solar radiation data are from EUMETSAT’s Satellite Application Facility on Climate Monitoring (CM SAF) for the years 1983–2016^60^. Summary of the datasets used in this study are displayed in Table 1.

**Table 1:** Observation data and model data used in the study.

Observation Data	Source
Temperature	Atmospheric Infrared Sounder (AIRS)^55^ and ERA-Interim^56^
Relative humidity	Atmospheric Infrared Sounder (AIRS)^55^ and ERA-Interim^56^
Precipitation	Global Precipitation Climatology Project (GPCP)^58^ v2.3
Shortwave radiation	EUMETSAT^60^ CM-SAF based on SEVIRI sensors
Surface fluxes	ERA-Interim
Cloud cover fraction	EUMETSAT^60^ CM-SAF based on SEVIRI sensors
Geopotential height at 700 mb	NCEP-NCAR^59^ reanalysis
**Model Data**	**Source**
Global and regional scale historical simulations (ALL forcing)	19 CMIP5^18^, 2 CORDEX^61^
Global and regional scale scenario simulations (RCP4.5)	19 CMIP5, 2 CORDEX
Pre-industrial control simulations (12,000-year)	CMIP5
GHG-forcing only, AA-forcing only and LU-forcing only runs	CMIP5, 6^66^ (Historical-Misc.)
Paleo simulations (0850–1850 millennium)	CCSM4^62^ Paleo simulations

### Climate model data

Historical single forcing experiments (GHG-only, LU-only and AA-only) are based on fully coupled Earth System Models (ESMs) and future climate projections are from the Coupled Model Intercomparison Project Phase 5 (CMIP5) archive^18^. In order to address finer scale features of climate change signals, we also use data from the RCA4 regional climate model driven by MPI-ESM-LR and ICHEH models from the WCRP CORDEX^61^ project. In addition, we included 12,000-year Pre-industrial control simulations of CMIP5 models and the Millennium simulations over 0850–1850 performed with the fourth version of the Community Climate System Model (CCSM4)^62^ to obtain an estimate of natural variability of VPD. Prior to the attribution climate model verification has been applied (see Supp. Text S1).

### Calculating VPD

To calculate VPD we use the following equation (Eq. 1) based on monthly near surface air temperature (*T*) and dew point (*Td*)^25^^,^^27,7^.

1 $$VPD={c}_{1}\times \exp (\frac{{c}_{2}\ast T}{{c}_{3}+T})-{c}_{1}\times \exp (\frac{{c}_{2}\ast {T}_{d}}{{c}_{3}+{T}_{d}})$$

Where, c_1_ = 0.611 KPa, c_2_ = 17.5, c_3_ = 240.978 °C. *T* and *Td* are in °C and VPD is in KPa. The first and the second term in Eq. 1 are the saturation vapor content of air *T* (***e***_***s***_) and the actual vapor pressure (***e***_***a***_), respectively. Temperature (*T*) and relative humidity (*RH*) are used to calculate dew point (*Td*) in climate models based on the following equation (Eq. 2):

2 $${T}_{d}=\frac{{a}_{1}\ast \{\mathrm{ln}(\frac{RH}{100})+\frac{{a}_{2}\ast T}{{a}_{1}+T}\}}{{a}_{2}-\{\mathrm{ln}(\frac{RH}{100\,})+\frac{{a}_{2}\ast T}{{a}_{1}+T}\}}$$

Where, a_1_ = 243.04, a_2_ = 17.625.

### Estimating anthropogenic climate change signals

We used two approaches for the estimation: **1**. The historical single-forcing experiments from the CMIP5 archive were organized in 3 groups. One group (GHG) includes 7 models forced with historical well-mixed greenhouse gas only. A second group (LU) includes 3 models forced with land-use change only. A third group (AA) includes 4 models forced with anthropogenic aerosols only. We further subdivided the AA simulations into two groups to separately investigate the impact of aerosol concentration on, (1) “cloud albedo effect” or “first indirect effect”^33^, and (2) “cloud lifetime effect”^33^ or “second indirect effect”. Hence, AA1 simulations included both the first and second indirect effect, while AA2 simulations included the first indirect effect only.

**2**. We used the time-slice climate change experiments and defined the GS signal (Greenhouse gas and Sulfate aerosols, GS) as the difference between the last decades of the 21st century (2071–2100, RCP4.5 scenario) and the reference climatology (1961–1990), and scaled the resulting signal to change per year^63^. In total, we used 19 models from CMIP5 archive and 2 models from CORDEX. The models and the number of runs with each model are presented in Table S1.

The formula to calculate effective number of models with equal weighting of the individual models is:

3 $$n=\frac{{d}^{2}}{{\sum }_{i=1}^{d}\frac{1}{{l}_{i}}}$$

where d is the number of models and l is the ensemble size^64^. The final internal variance is then just 1/n the internal variance.

### Signal attribution

The attribution analysis here is based on estimating the amplitude of the response of VPD to each external forcing from the observations via the estimation of scaling factors^28,29^. In order to account for the noise in response patterns Total Least Squares (TLS) methodology^65^ is used:

4 $$y-{u}_{0}=\mathop{\sum }\limits_{i=1}^{m}{R}_{i}({x}_{i}-{u}_{i})$$

where y represents the observations and each x_i_ the modeled response to one of *m* forcings that is anticipated by climate models. *R*_*i*_ is an unknown scaling factor. The noise on *y*, denoted by *u*_*0*_, is assumed to represent internal climate variability, while the noise on *x*_*i*_, denoted by *u*_*i*_, is a result of both internal variability and the finite ensemble used to estimate the model response. We examine the null hypothesis that the observed change in VPD is drawn from a hypothetical population of a climate disturbed by a specific (m) external influence. Attribution is claimed in cases where the scaling factors and associated internal variability-generated uncertainties are inconsistent with zero but consistent with one.

### Bowen ratio and evaporative fraction

The Bowen ratio is defined as the ratio of sensible heat flux (*Q*_*s*_) divided by latent heat flux (*Q*_*l*_). The Evaporative Fraction (EF) is the ratio of latent heat flux (*Q*_*l*_) to surface available energy.

5 $$Bowen\,ratio=\frac{{Q}_{s}}{{Q}_{l}}$$

6 $$Evaporative\,Fraction=\frac{{Q}_{l}}{{Q}_{s}+{Q}_{l}}$$

### Linear regression model

To represent the land-atmosphere interactions, we used the Bowen ratio as a measure for energy partitioning, and we chose the geopotential height at 700 mb where moisture transport is maximum for the atmospheric circulation. The following regression model was developed:

7 $$VPD(t)=a\ast Temp(t)+b\ast Bowen(t)+c\ast Hg{t}_{700}(t)+d$$

where *t* is time. The predictand VPD, and the predictors Temperature, Bowen ratio and Hgt_700_ are normalized (i.e., minus mean and divided by the standard deviation). The constants *a*, *b* and *c are* fitted to the data. We apply our regression model in two regions: the northwestern Amazonia covered by dense tropical forest (with high climatological rainfall (~180 mm) and low climatological VPD (~10 mb), Fig. S5) and the southeastern Amazonia mostly covered by tropical savanna (with low rainfall (~90 mm) and high VPD (~18 mb) climatology, Fig. S5).

## Supplementary information

Supplementary material
