# Supplementary material for: A Recent Systematic Increase in Vapor Pressure Deficit over Tropical South America
Source: Sci Rep. 2019 Oct 25;9:15331. doi: 10.1038/s41598-019-51857-8 (PMC6814800; doi:10.1038/s41598-019-51857-8)
Supplement: Supplementary file 1 — Supplementary material [file 41598_2019_51857_MOESM1_ESM.pdf]

Supporting Information

A recent systematic increase in vapor pressure deficit over tropical South America

Armineh Barkhordarian *[1,2], Sassan S. Saatchi[2,3,] Ali Behrangi[4], Paul C. Loikith[5], Carlos R. Mechoso[1]

[1]*Department of Atmospheric and Oceanic Sciences. University of California, Los Angeles (UCLA)*
[2]*Jet Propulsion Laboratory, California Institute of Technology, Pasadena, USA.*
[3]*Institute of Environment and Sustainability, University of California, Los Angeles, CA, USA*
[4]*University of Arizona, Department of hydrology and atmospheric sciences, Tucson, USA.*
[5]*Portland State University, Department of Geography, Portland Oregon, USA.*

Corresponding author: Armineh Barkhordarian (barkhora@g.ucla.edu)

This PDF file includes:

Table S1. List of models and number of simulations from CMIP5 experiments.
Text S1. Evaluation of Climate models:
Fig. S1. Daytime and nighttime trends in VPD based on AIRS Satellite data.
Fig. S2. Daytime and nighttime trends in Relative Humidity based on AIRS Satellite data.
Fig. S3. Changes of VPD based on ERA5 reanalysis data.
Fig. S4. Detection of externally forced changes in VPD trends in wet months (March-May, MAM)
Fig. S5. Climatology pattern of VPD (AIRS) and Climatology pattern of Rainfall (TRMM)
Fig. S6. Seasonal cycle of VPD over 2003-2016 based on AIRS satellite data
Fig. S7. Spatial map of model biases in simulating the observed annual trends in VPD
Fig. S8. Simulated response pattern of VPD to external drivers (GHG, LU and AA1)
Fig. S9. Time series in cloud cover anomalies from EUMETSAT (CM-SAF)

Table S1: list of models and number of simulations from CMIP5 experiments. (Taylor et al., 2011)

	**Institute ID**	**Models (number of runs)**
1	CSIRO-BOM	ACCESS1-0 **(1)**
2	CSIRO-BOM	ACCESS1-3 **(1)**
3	BCC	BCC-CSM1-1-M **(4)**
4	BCC	BCC-CSM-1 **(4)**
5	NCAR	CCSM4 **(5)**
6	CNRM-CERFACS	CNRM-CM5 **(5)**
7	NOAA GFDL	GFDL-CM3 **(5)**
8	NASA GISS	GISS-E2-H-CC **(1)**
9	NASA GISS	GISS-E2-H **(4)**
10	NASA GISS	GISS-E2-R-CC **(1)**
11	NASA GISS	GISS-E2-R **(4)**
12	MOHC	HadGEM2-AO **(1)**
13	MOHC	HadGEM2-CC **(4)**
14	INM	INM-CM4 **(2)**
15	IPSL	IPSL-CM5A-LR
16	MIROC	MIROC-ESM-CHEM **(1)**
17	MIROC	MIROC-ESM **(3)**
18	MIROC	MIROC5 **(3)**
19	MRI	MRI-CGCM3 **(5)**

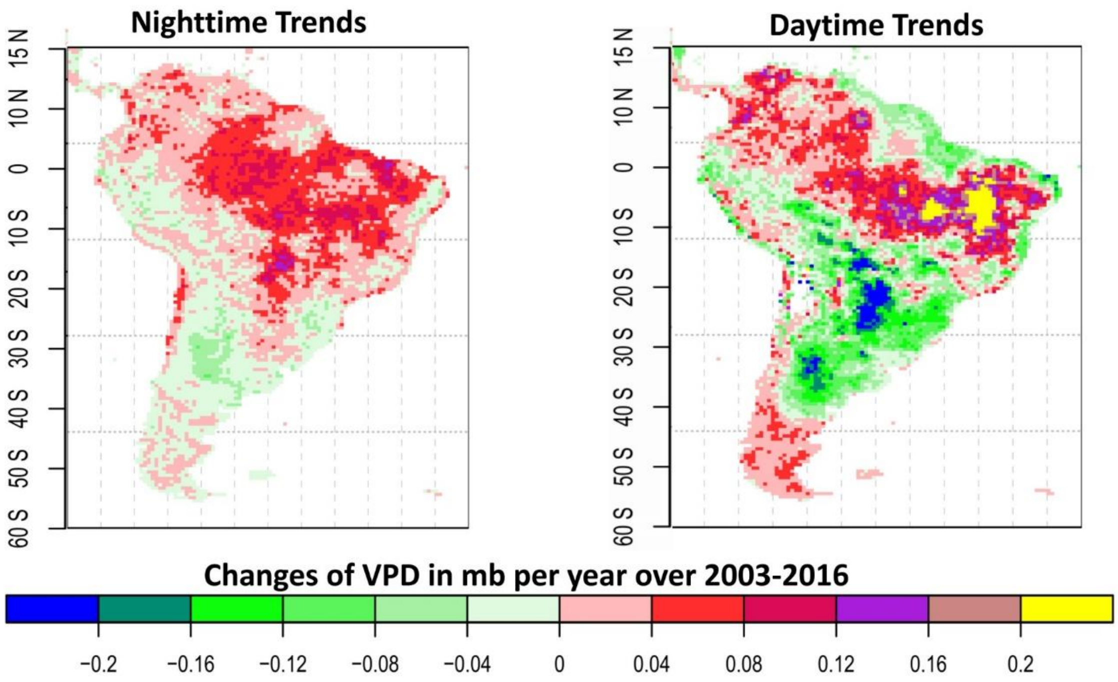

Figure S1: Daytime and nighttime trends in **Vapor Pressure Deficit** (VPD) over 2003-2016 based on AIRS Satellite data. Units are mb per year.

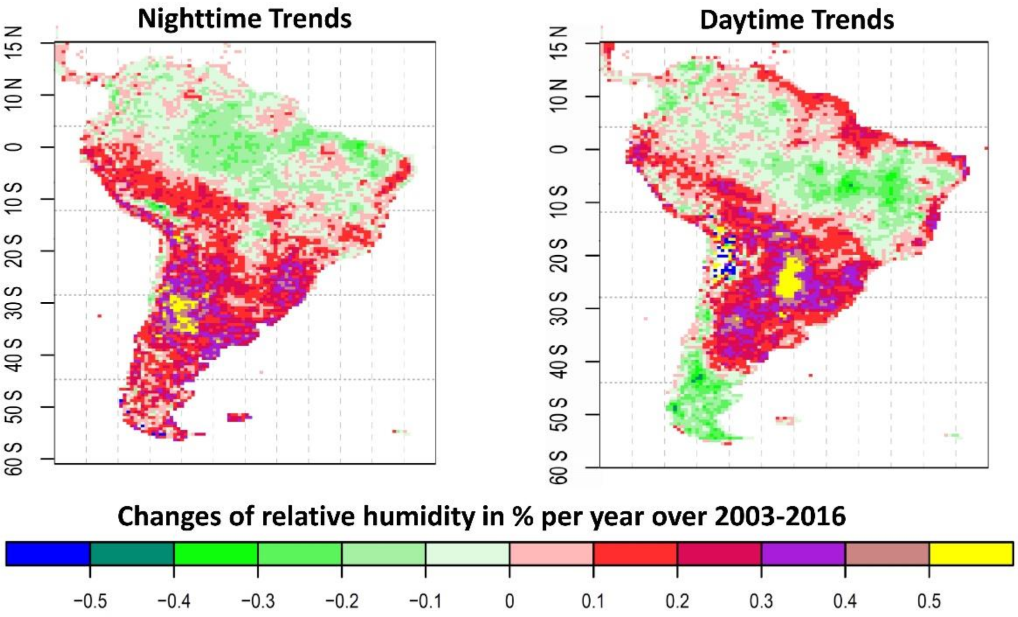

Figure S2: Daytime and nighttime trends in **Relative Humidity** over 2003-2016 based on AIRS Satellite data. Units are % per year.

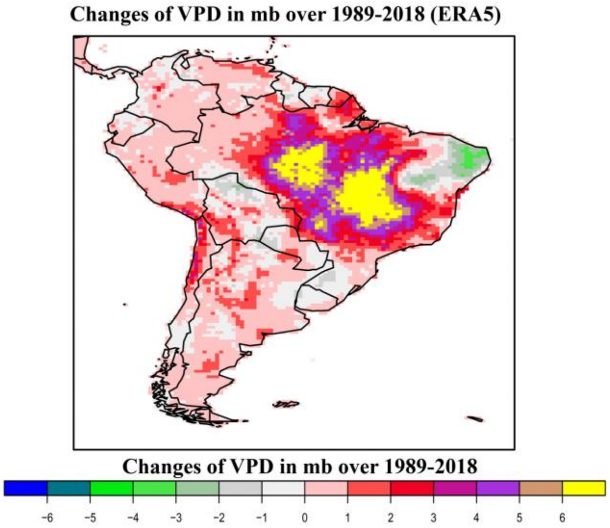

Figure S3: Changes of VPD in dry ASO (August-September-October) months in mb over 1989-2018 based on ERA5 reanalysis data (The fifth generation of ECMWF atmospheric reanalysis data). Units are mb in 30 year.

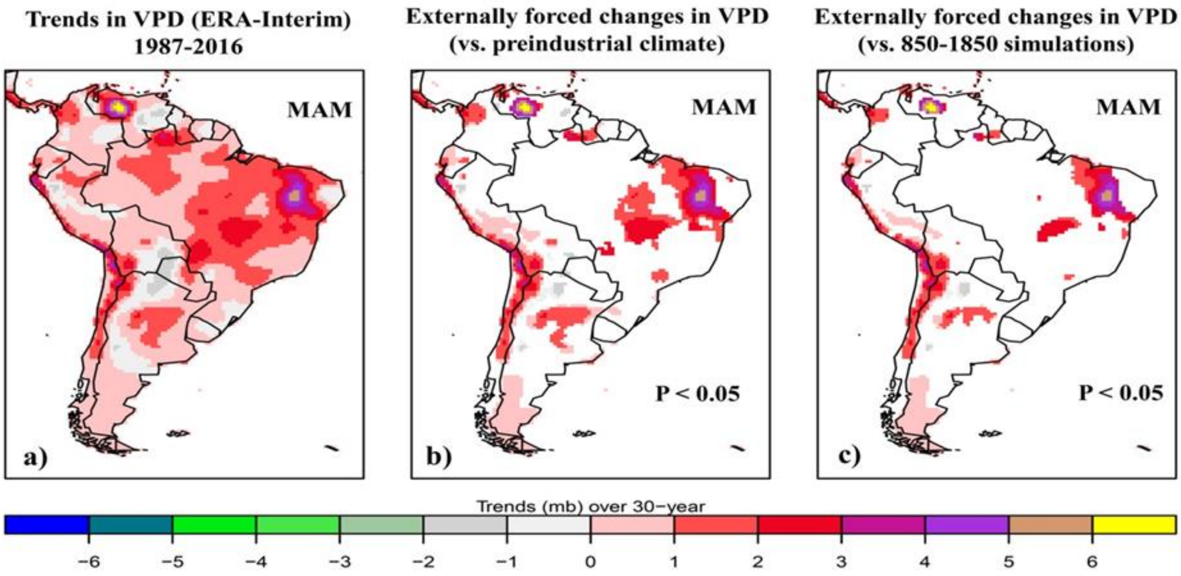

Figure S4: Detection of externally forced changes in VPD trends (MAM). a) Trends in VPD derived from ERA-I during 1987-2016 in wet (March-May, MAM) months. **b)** Regions where externally forced changes of VPD are detectable (P < 0.05) in MAM (in comparison with 400 pseudo-realizations of unforced trends derived from 12,000-year Pre-industrial simulations). **c)** Regions where anthropogenically forced changes of VPD are detectable (P < 0.05) MAM, (in comparison with 30 pseudo-realizations of naturally forced trends derived from Paleo simulations).

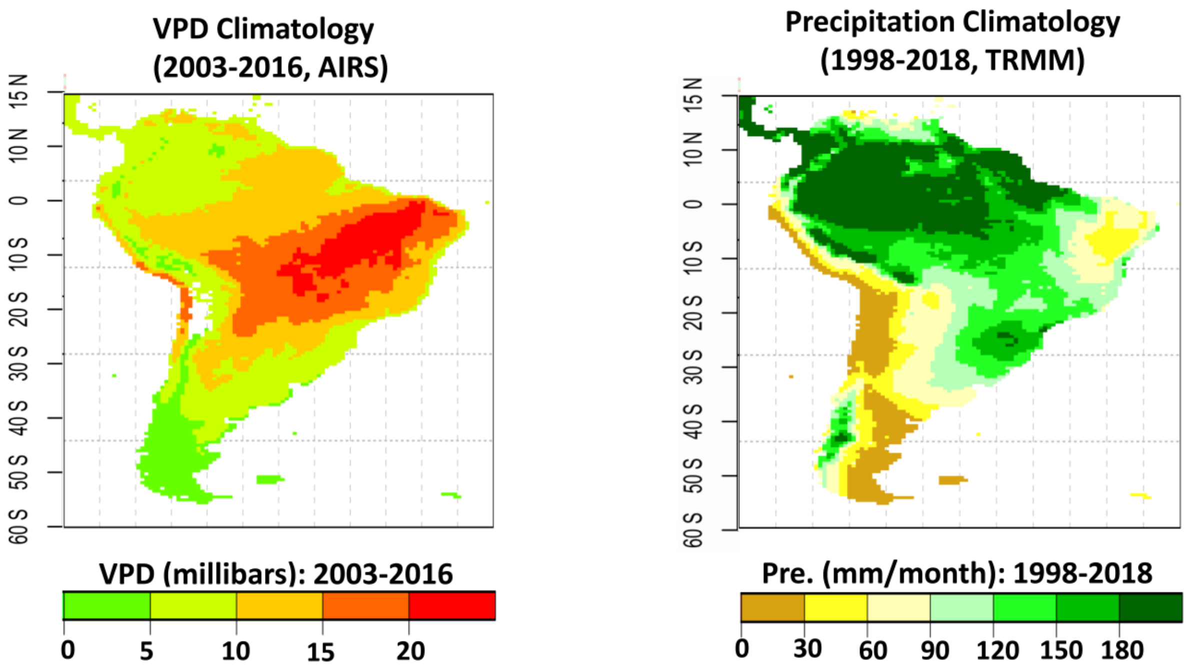

Figure S5: ***Left:*** Climatology of VPD (mb/month) over 2003 to 2016 based on AIRS satellite data**.** ***Right:*** Climatology of precipitation (mm/month) over 1998 to 2018 based on TRMM data.

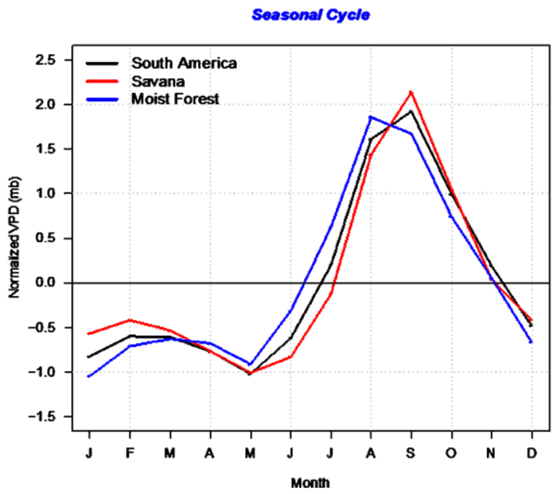

Figure S6: Seasonal cycle of VPD over 2003-2016 based on AIRS satellite data. Normalized VPD values (i.e., minus mean and divided by the standard deviation).

Text S1: Evaluation of Climate models:

In order to assess the performance of the climate models in simulating the observed changes in VPD a model verification analysis has been applied. Model biases are calculated using the historical (All forcing) runs of fully coupled Earth System Models (ESMs) that are used to estimate the response of VPD to the biophysical effect of Land-use/land-cover change.

Figure S7 displays the spatial map of model biases in simulating the observed annual trends in VPD ($\Delta M - \Delta O$). Negative (positive) values indicate that the models underestimate (overestimate) the observed changes. The areas where the magnitude of the biased trend is not significantly greater than zero is masked out in order to only focus on regions that the model bias is due to lack of local forcings and feedbacks, and not due to internal variability-generated uncertainties. As shown in Figure S7 models underestimate the observed VPD increase over eastern Brazil that extends to Paraguay and Dry Chaco region. These regions have high climatological VPD (Figure S5), biases could imply that models are less water limited than observation (Dunn et al., 2017). While we acknowledge the presence of theses model biases, we believe they do not affect the attribution statements in this study.

Figure S8 shows the observed trend pattern of VPD over 1983-2012 in comparison with the simulated response of VPD to GHG+AA1+LU forcing, greenhouse-gas forcing (GHG), anthropogenic aerosols forcing (AA1), and land-use change forcing (LU). The response of VPD to GHG forcing is pronounced over the entire Tropical South America. The increasing response of VPD to the effect of biomass burning aerosols are more pronounced over southeastern Brazil, Rondônia and Chaco regions, which are deforestation hotspots. With this trend pattern comparison, we conclude that the climate models, used in this study, are reasonably reproducing the observed changes of VPD in the region.

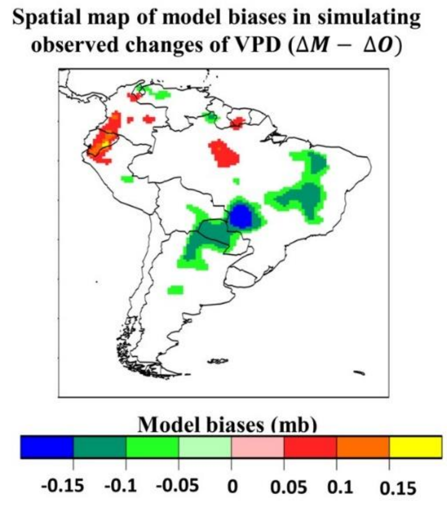

Figure S7: Spatial map of model biases in simulating the observed annual trends in VPD ($\Delta M - \Delta O$). Negative (positive) values indicate that the models underestimate (overestimate) the observed trends in VPD. The areas where the magnitude of the biased trend is not significantly greater than zero is masked out in order to only focus on regions that the bias is due to local forcing and/or feedbacks and not due to internal variability-generated uncertainties. Units are mb.

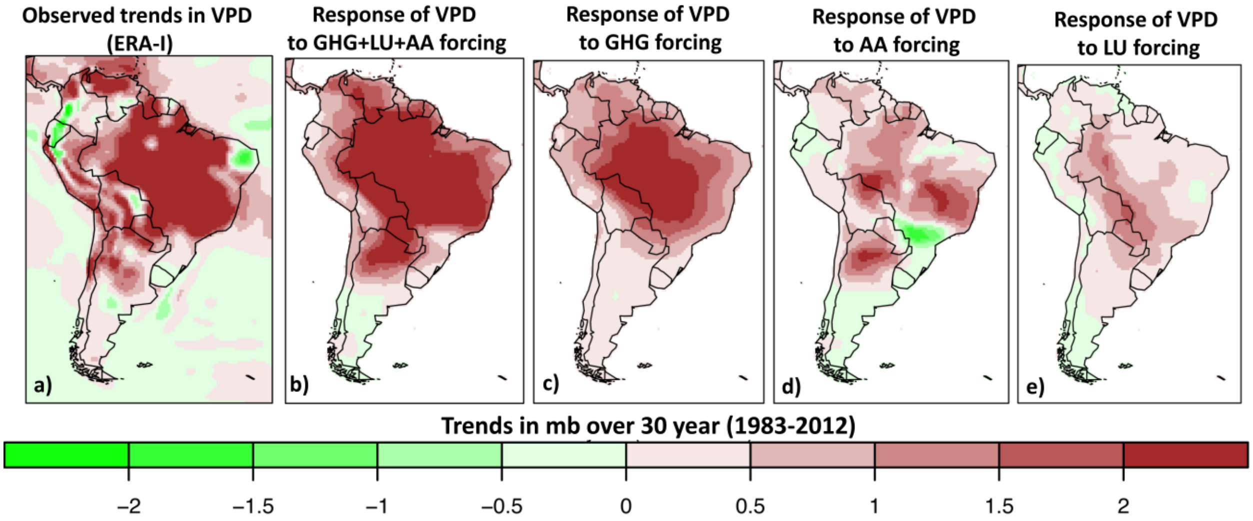

Figure S8: *Left to right:* (a) the observed trend pattern of VPD based n ERA-I (1983-2012) in comparison with the simulated response of VPD to (b) GHG+AA1+LU forcing, (c) greenhouse-gas forcing (GHG), (d) anthropogenic aerosols forcing (AA1), and (e) land-use change forcing (LU). Units are mb.

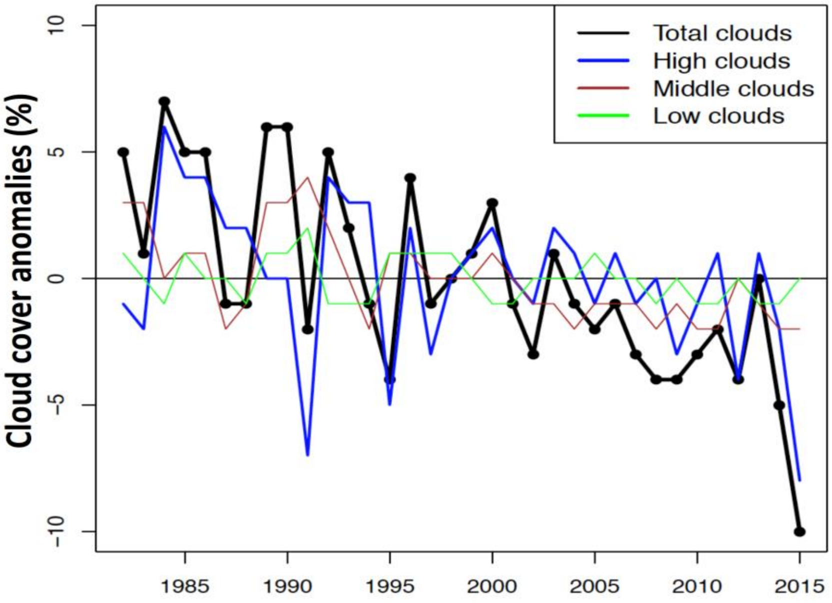

Figure S9: Time series in cloud cover anomalies from EUMETSAT (CM-SAF based on SEVIRI sensors) in ASO (August-October) over tropical South America over 1983-2015.
